# MicroRNA-378 protects against intestinal ischemia/reperfusion injury via a mechanism involving the inhibition of intestinal mucosal cell apoptosis

**DOI:** 10.1038/cddis.2017.508

**Published:** 2017-10-12

**Authors:** Yunsheng Li, Shihong Wen, Xi Yao, Weifeng Liu, Jiantong Shen, Wentao Deng, Jing Tang, Cai Li, Kexuan Liu

**Affiliations:** 1 Department of Anesthesiology, Nanfang Hospital, Southern Medical University, Guangzhou, 510515 China; 2 Department of Anesthesiology, The First Affiliated Hospital, Sun Yat-sen University, Guangzhou 510080, China; 3 Department of Anesthesiology, Shaanxi Provincial People’s Hospital, Shaanxi 710068, China

## Abstract

Intestinal ischemia/reperfusion (I/R) injury remains a major clinical event and contributes to high morbidity and mortality rates, but the underlying mechanisms remain elusive. Recent studies have demonstrated that microRNAs (miRNAs) have important roles in organ I/R injury, but the changes and potential roles of miRNAs in intestinal I/R-induced intestinal injury are unclear. This study was designed to analyze the miRNA expression profiles in intestinal mucosa after I/R injury and to explore the role of target miRNA during this process. Using miRNA microarray analysis, we found changes of 19 miRNAs from the expression profile of miRNAs in a mouse model of intestinal I/R and further verified them by RT-qPCR. Here, we report that miR-378 is one of the markedly decreased miRNAs and found the putative target mRNA that is linked to cell death after applying the TargetScan, miRanda, CLIP-Seq and miRDB prediction algorithms. Our results show that the overexpression of miR-378 significantly ameliorated intestinal tissue damage in wild-type and transgenic mice and oxygen glucose deprivation/reperfusion-challenged IEC-6 cell injury. Moreover, miR-378 overexpression reduced intestinal epithelial cell apoptosis in both *in vivo* and *in vitro* ischemic models and attenuated cleaved caspase-3 expression. Collectively, our results revealed that the suppression of caspase-3 activation by miRNA-378 overexpression may be involved in the protective effects of intestinal ischemic damage. MiRNA-378 may serve as a key regulator and therapeutic target in intestinal I/R injury.

Intestinal ischemia/reperfusion (I/R) injury is a potentially serious consequence of acute mesenteric ischemia, hemorrhagic, traumatic or septic shock, severe burns or some surgical procedures, including small bowel transplantation and aortic aneurysm repair.^1^ Intestinal I/R not only leads to injury of the intestine itself, but also involves severe destruction of distant tissues because of disruption of the intestinal mucosal barrier, which is associated with local and systemic injuries that ultimately progress to multiple organ dysfunction and often death.^2^ Therefore, the development of effective treatment strategies against intestinal I/R is important for improving the prognosis of critically ill patients.

The factors contributing to intestinal I/R injury were complex, including microvascular dysfunction,^3^ reactive oxygen species over-production,^4, 5^ inflammation^6, 7^ and even intestinal epithelial cell death. Many evidence reveals that apoptosis is a major mode of cell death caused by intestinal I/R,^8, 9^ which is a complex biological process that can be triggered by the death receptor and mitochondrial death signaling pathways.^10^ The small intestine is prone to be subjected to ischemic-induced apoptosis because of the priority of blood flow supplied to brain and heart when hypovolemic shock occurs. Various studies demonstrated that prophylactic anti-apoptotic treatment could be an effective therapeutic strategy for the prevention of intestinal I/R injury,^11, 12, 13^ including pharmacological blockade of protein kinase C *β*2,^14^ PI3K/AKT pathway activation^15^ and so on. However, the underlying molecular mechanism of I/R-induced intestinal epithelium apoptosis is remained poorly understood and effective pharmacological or genetic agents would be rational to develop.

MicroRNAs (miRNAs) are a novel class of endogenous, small non-coding single-stranded RNAs, with highly conserved sequences among species. Through imperfect sequence-specific binding to the 3’-untranslated region (UTR) of target messenger RNAs (mRNAs), miRNAs downregulate gene expression by degrading target mRNAs and/or inhibiting protein synthesis.^16, 17^ Meanwhile, it has been found to be not only crucial for the development and maintenance of physiological homeostasis, but have also been causally implicated in tissue injury and repair.^18, 19, 20^ Recent studies showed that characteristic changes of miRNAs have important roles in cardiac,^21^ cerebral,^22^ renal^23^ I/R injury, which are associated with cell apoptosis, oxidative stress and inflammation. Meanwhile, emerging evidence suggests that miRNAs are of paramount importance in gastrointestinal development and physiology.^24, 25^ However, the differential miRNAs expression of intestinal mucosa in response to intestinal I/R are still unclear, and the contributions of miRNAs to post-ischemic intestine remain to be elucidated.

Thus, studies are needed to better understand the characterization of miRNAs expression and their function involved in intestinal I/R-induced injury. In this study, we aimed to analyze miRNAs expression profiles in intestinal mucosa after I/R injury and explore the role and potential mechanisms of target miRNAs on intestinal epithelial cell apoptosis induced by intestinal I/R injury.

## Results

### Evaluation of intestinal I/R injury

**Figure 1:**
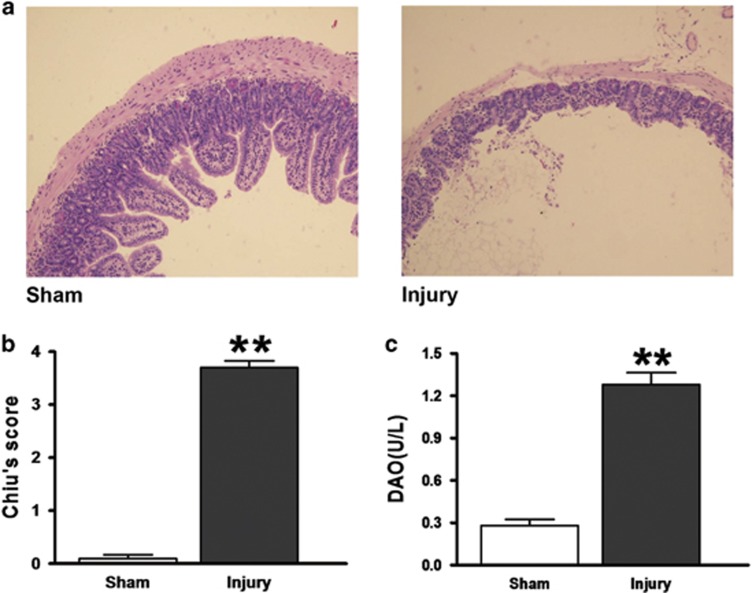
Evaluation of intestinal injury induced by intestinal I/R. Male FVB/N mice (8–10 weeks old) underwent a sham operation or SMA occlusion for 60 min followed by 120-min reperfusion. (**a**) Histopathologic changes of the intestinal mucosa (hematoxylin and eosin staining, magnification × 100). The intestinal mucosa was intact in the sham group, whereas severe edema of the mucosal villi and infiltration of inflammatory cells were observed in the Injury group. (**b**) Injury scores for the intestinal mucosa morphology. (**c**) Intestinal cellular injury evaluated by serum DAO activity. The data are expressed as the mean±S.D. (*n*=8). **P< 0.01 compared with the Sham group

Representative intestine sections (*n*=8 per group) revealed that 60 min of ischemia and 120 min of reperfusion caused significant intestinal mucosal damage that was primarily manifested as severe edema of the mucosal villi, infiltration of inflammatory cells and increased gaps between epithelial cells. By contrast, normal mucosal architecture was observed in the sham-operated mice (Figure 1a). Consistent with the histological changes in the intestinal mucosa, Chiu’s score, which reflects the severity of histological damage, was significantly higher in the injury group than in the sham group (Figure 1b). Moreover, the plasma diamine oxidase (DAO) levels in the injury group were markedly higher than those in the sham group (Figure 1c). This well-established model was used in the subsequent experiments.

### Identification of downregulated miR-378 in the intestines of mice following intestinal I/R

MiRNA microarray analysis revealed that 19 differentially expressed miRNAs were identified in response to intestinal I/R, including 1 upregulated and 18 downregulated miRNA candidates, all of which exhibited a *P*-value <0.05 calculated by *t*-test when compared with the sham group (*n*=5 per group, Table 1). The above differential miRNA expression levels in the ischemic intestine were further validated by RT-qPCR. To characterize intestinal I/R-responsive miRNA candidates, the following parameters were defined: a fold change of at least 1.5, and putative target mRNAs that were linked to cell death as identified using the prediction algorithms TargetScan, miRanda, CLIP-Seq and miRDB. Consequently, miR-378 was found to exhibit a significant fold change (3.38-fold downregulation, validated by RT-qPCR) among the I/R-induced miRNAs (Table 2). Moreover, among the 40 predicted putative targets of miR-378, three are common to the above four algorithms, including MTSS1L, NEUROD1 and CASPASE-3 (Table 3).

### MiR-378 agomir protects against ischemic intestinal injury and cell apoptosis in the wild-type (WT) mouse intestinal I/R model

**Figure 2:**
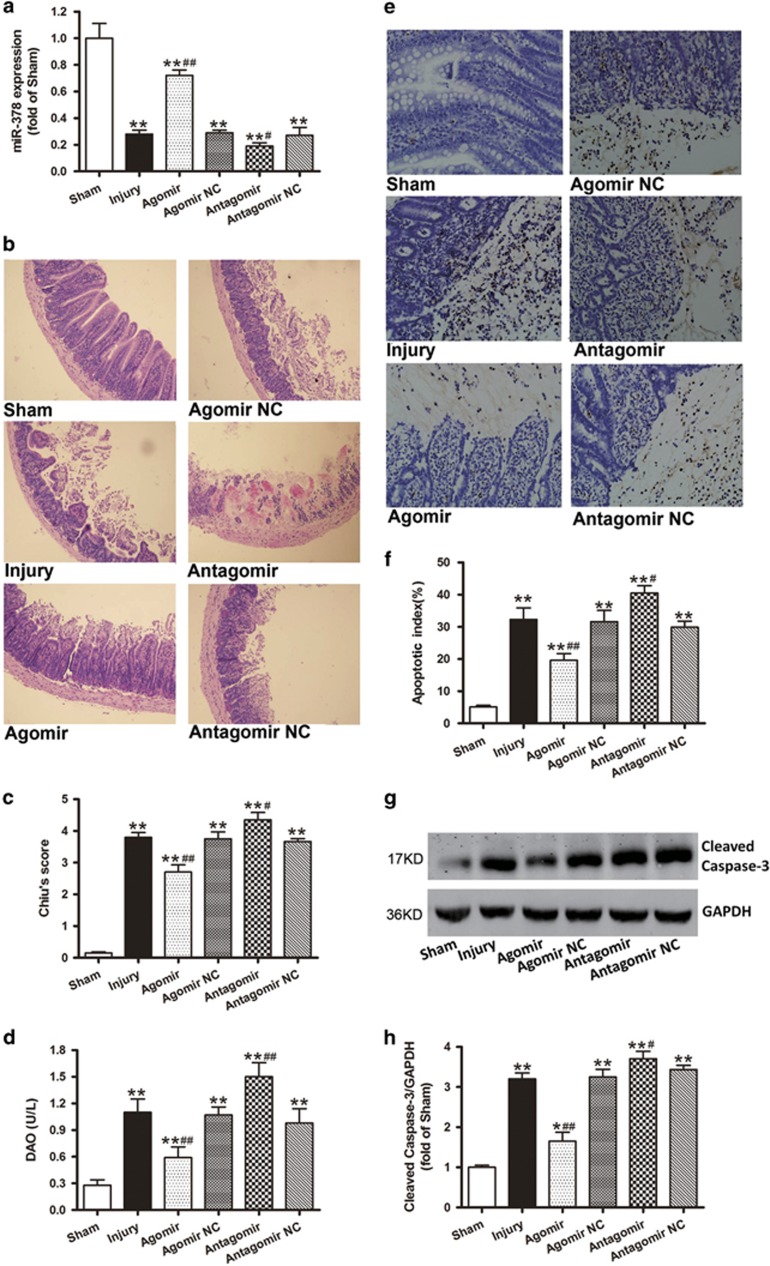
MiR-378 agomir protects against ischemic intestinal injury and cell apoptosis in the WT mouse. The mice received injections of agomiR-378, antagomiR-378 or their NCs (100 *μ*l) via the tail vein (40 mg/kg body weight) for 3 consecutive days. The intestinal I/R injury model was established on the fourth day after injection. (**a**) Changes of miR-378 expression levels after the administration of agomir, antagomir and their NCs. (**b**) Histopathologic changes of the intestinal mucosa (hematoxylin and eosin staining, magnification × 100). (**c**) Injury scores for the intestinal mucosa morphology. (**d**) Intestinal cellular injury evaluated by serum DAO activity. (**e** and **f**) Representative TUNEL staining and the quantifications of the apoptotic indices. The apoptotic nuclei are stained dark brown. Notable destruction of the villi and apoptotic enterocytes is present in the upper regions of the villi in the injury, agomir NC, antagomir and antagomir NC groups. Fewer detached epithelial cells with dark brown nuclei were observed in the enteric cavity of the agomir group. (**g** and **h**) Western blot analyses showing that the administration of agomiR-378, but not its NC, downregulated the caspase-3 cleavage. The data are expressed as the mean±S.D. (*n*=8). *P<0.05, **P<0.01 compared with the sham group; ^#^*P*<0.05, ^##^*P*< 0.01 compared with injury group

Three days after injection of agomir-378, antagomir-378 or their negative control (NC), miR-378 expression was significantly preserved in the agomir group compared with the other ischemic insult groups. Moreover, miR-378 expression in the antagomir group was significantly lower than that in the injury group (Figure 2a). In addition, the WT mice that received agomiR-378 exhibited slight edema and mucosal architecture destruction, and the damage was further ameliorated compared with injury group. Conversely, the intestinal injury induced by I/R was further aggravated after receiving antagomiR-378, as evidenced by increased Chiu’s scores and DAO levels (Figures 2b and d).

As shown in Figure 2e, the ileal tissues demonstrated the marked appearance of dark brown (TUNEL positive) apoptotic cells from the detached epithelium at the tips to the lower part of the villi in the injury mice. However, fewer positive-staining cells were observed in the ileum sections taken from the agomir mice. The intestinal mucosa apoptotic index was significantly reduced after pretreatment with agomir-378. By contrast, the antagomiR-378 treatment increased the number of apoptotic cells (Figure 2f). Consistently, pretreatment with agomir-378 resulted in a significant reduction in cleaved caspase-3 expression (Figures 2g and h).

### *In vivo* overexpression of miR-378 ameliorates the intestinal mucosal injury and cell apoptosis induced by intestinal I/R

**Figure 3:**
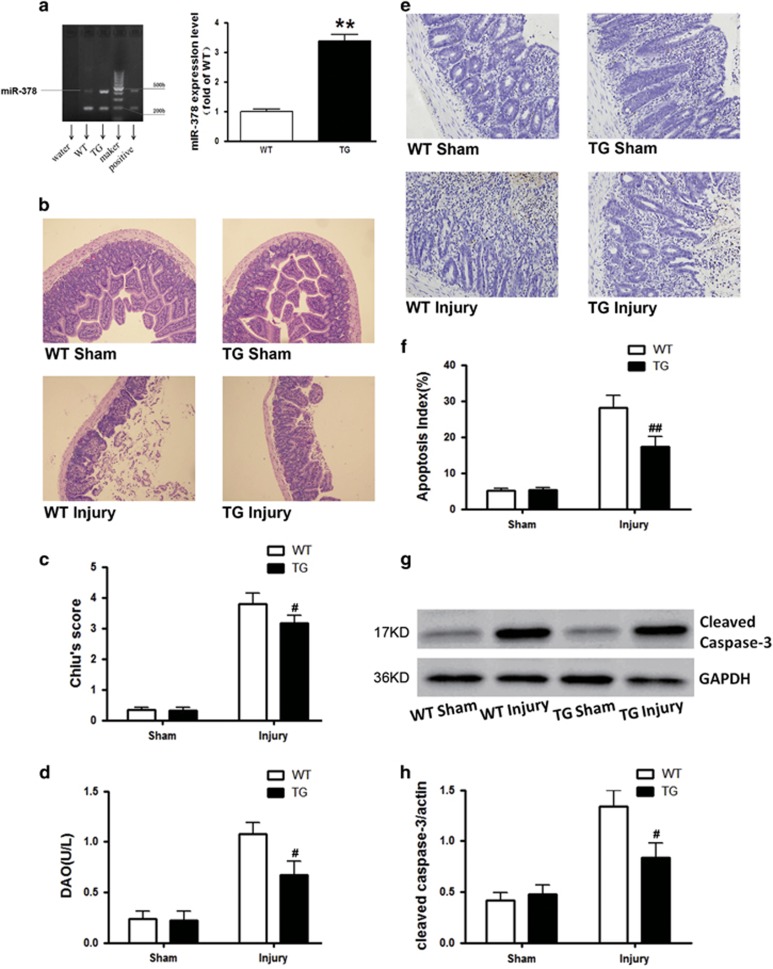
*In vivo* overexpression of miR-378 ameliorates the intestinal injury and cell apoptosis induced by intestinal I/R in TG mice. TG mice and WT mice were subjected to intestinal I/R injury. (**a**) RT-qPCR showed that the expression of miR-378 in TG mice was 3.39 times higher than that in the WT mice. (**b**) Histopathologic changes of intestinal mucosa in WT and TG mice subjected to I/R insult (hematoxylin and eosin staining, magnification × 100). (**c**) Injury scores for the intestinal mucosa morphology. (**d**) Intestinal cellular injury evaluated by serum DAO activity. (**e** and **f**) Representative TUNEL staining and the quantification of the apoptotic index. More apoptotic epithelial cells were observed at the villi in the WT injury group than in the miR-378 overexpression TG injury group. (**g** and **h**) Western blot analyses showing that overexpression of miR-378 in the TG mice led to lower cleaved caspase-3 expression after I/R insult. The data are expressed as the mean±S.D. (*n*=8). **P<0.01 compared with WT sham group, ^#^*P*<0.05, ^##^*P*<0.01 compared with WT injury group

The miR-378 level was detected by RT-qPCR to confirm the construction of the miR-378 transgenic (TG) mouse model. As illustrated, the expression of miR-378 in the TG mouse was 3.39 times that in the WT mice (Figure 3a). Compared with the WT mice, the intestinal injury induced by intestinal I/R in the TG mice was mitigated as evidenced by the improved histological injury and decreased DAO levels (Figures 3b and d). Moreover, as illustrated in Figure 3e, the ileal tissues demonstrated the marked appearance of dark brown (TUNEL positive) apoptotic cells from the detached epithelium at the tips to the lower part of the villi in the WT injury mice. However, fewer positive-staining cells were observed in the ileal sections taken from the TG Injury mice. The apoptotic index of WT injury group was markedly higher than that of the TG injury group (Figure 3f). Moreover, the significant reduction in epithelial apoptosis correlated with lower cleaved caspase-3 expression in the TG injury mice (Figures 3g and h).

### *In vitro* overexpression of miR-378 improves IEC-6 cell survival after OGD/R challenge

**Figure 4:**
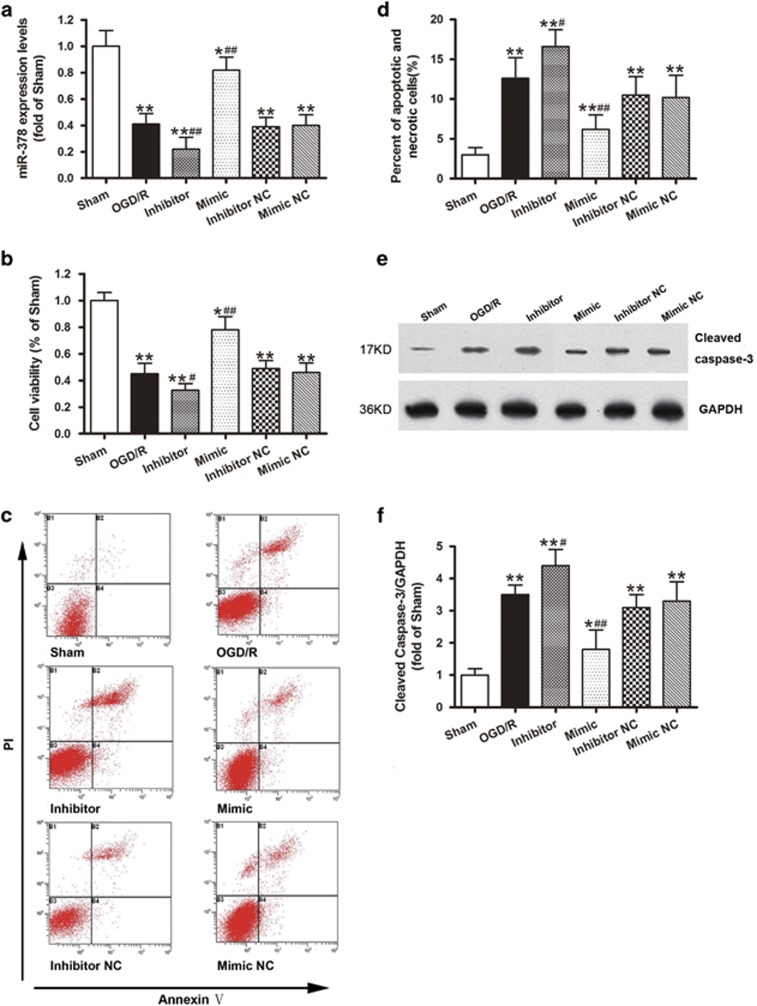
*In vitro* overexpression of miR-378 improves IEC-6 cell survival and inhibits caspase-3 activation after OGD/R challenge. (**a**) MiR-378 expression levels after transfection of miR-378 mimic, miR-378 inhibitor or their NCs. (**b**) Cultured IEC-6 cell injury was induced by depriving the culture media of oxygen and glucose (OGD), and cell viability was measured by MTT after transfection. (**c**) Representative Annexin V/PI dot plots of the flow cytometry analysis. The percentages of cells in the right upper and right lower quadrant (Annexin V-positive-staining cells represent apoptosis and necrosis) in each plot were analyzed. (**d**–**f**) MiR-378 mimic transfection resulted in significant downregulation of cleaved caspase-3 when compared with the OGD/R group, whereas pretreatment with miR-378 inhibitor further increased the cleaved caspase-3 expression. The data are expressed as the mean±S.D. (*n*=6). *P<0.05, **P<0.01 compared with the sham group; ^#^*P*<0.05, ^##^*P*<0.01 compared with OGD/R group

As illustrated in Figure 4a, the transfection of the pre-miR-378 (mimic) or miR-378 inhibitor, but not their NCs, significantly increased or decreased the miR-378 levels in the normoxic cells. Cell viability analysis using the MTT assay revealed that 4 h of OGD followed by 4 h of reoxygenation resulted in obvious cell death, whereas pre-miR-378 treatment markedly augmented cell survival after OGD/R challenge. Moreover, pretreatment with the miR-378 inhibitor further exacerbated cell death when compared with the OGD/R group (Figure 4b). Flow cytometric analysis was used to further confirm whether miR-378 could affect 4 h OGD/4 h reoxygenation-induced IEC-6 cell apoptosis. As shown in the dual staining (Figure 4c), Annexin V^+^/PI^+^ and Annexin V^+^/PI^-^ (quadrants 2 and 4 represent apoptosis and necrosis) cells were rarely seen in the sham group, whereas OGD/R increased the numbers of apoptotic and necrotic cells. Transfection with pre-miR-378 could significantly reduce, whereas the miR-378 inhibitor aggravated, the numbers of apoptotic and necrotic OGD/R cells (Figure 4d). Consistent with the flow cytometric analysis, miR-378 mimic transfection resulted in a significant downregulation of cleaved caspase-3 when compared with the OGD/R group. Furthermore, pretreatment with the miR-378 inhibitor further increased the cleaved caspase-3 expression (Figures 4e and f).

### MiR-378 reduces caspase-3 expression in the intestine by targeting the 3’-UTR of caspase-3

**Figure 5:**
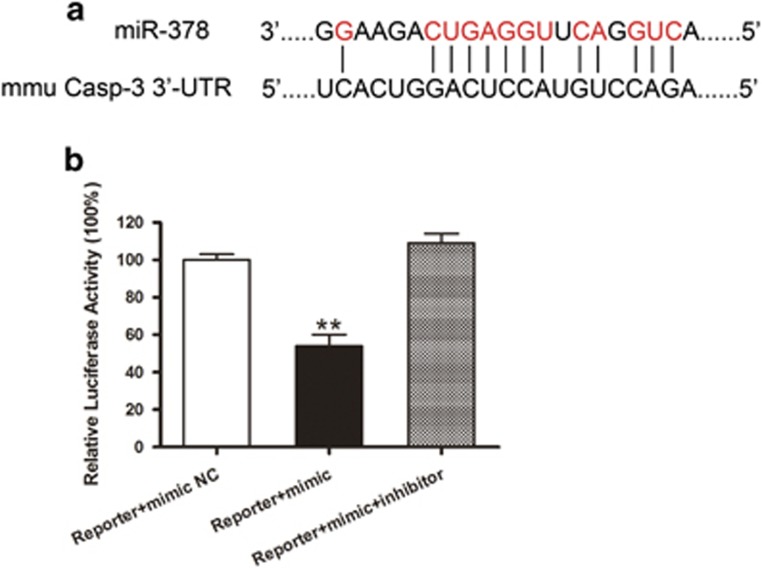
The luciferase reporter assay of miR-378. MiR-378 mimic, miR-378 inhibitor and NC mimic were co-transfected with a modified control vector containing the caspase-3 3’-UTR. (**a**) A schematic representation of the interaction between miR-378 and the 3’-UTR of caspase-3. (**b**) The luciferase assay showed that miR-378 downregulated 46% of the expression of caspase-3 in the Reporter+mimic miR-378 group, whereas the miR-378 inhibitor reversed the effect of miR-378. The results indicated that miR-378 acts on the 3’-UTR. The data are expressed as the mean±S.D. (*n*=5 independent experiments). **P<0.01 compared with the Reporter+mimic NC group

Caspase-3, a critical executor of apoptosis, is one of the predicted putative targets of miR-378 based on the prediction algorithms. One miR-378-binding site was identified within the 3’-UTR of the caspase-3 mRNA (Figure 5a). A dual-luciferase reporter assay was performed to validate caspase-3 as a putative target of miR-378. The co-transfection of 293 T cells with caspase-3-luc and a miR-378 mimic reduced the expression of luciferase by 46% compared with the results following NC caspase-3-luc/miR-378 mimic co-transfection (Figure 5b). These results indicated that miR-378 decreased the expression of caspase-3 through its direct binding with the caspase-3 3’-UTR.

## Discussion

Intestinal I/R is a serious clinical event that is associated with high morbidity and mortality. This study presented two important findings. First, with a miRNA microarray approach, we searched for miRNAs that were differentially expressed in the intestinal mucosa in response to intestinal I/R. We identified 18 downregulated miRNAs with expression that was decreased at least 1.5-fold. MiR-378 is one of these markedly decreased miRNAs and was found to be the putative target mRNA linked to cell death based on the application of the TargetScan, miRanda, CLIP-Seq and miRDB prediction algorithms. Furthermore, we demonstrated that the overexpression of miR-378 significantly ameliorated intestinal tissue damage in WT/TG mice and OGD-challenged IEC-6 cell injury. Second, overexpression of miR-378 reduced intestinal epithelial cell apoptosis in both *in vivo* and *in vitro* ischemic models and attenuated cleaved caspase-3 expression. These results imply that the protective effects of miR-378 following intestinal ischemia are likely mediated by the inhibition of cell apoptosis via the translational repression of caspase-3.

MiRNAs are known to be important mediators of gene regulation in response to cell-to-cell signaling and to act in the negative feedback of gene regulation,^16, 17^ which affects several biological processes, such as development,^26^ differentiation,^27^ apoptosis^28, 29, 30^ and oncogenesis.^31^ MiRNA analyses in organ I/R injury have been reported in recent studies. Xu *et al.*^32^ found 78 miRNAs that exhibit more than twofold differences in the liver upon I/R injury. Among these miRNAs, four miRNAs, including miR-23a, miR-326, miR-346_MM 1 and miR-370, were further significantly downregulated by ischemic preconditioning compared with the levels in non-preconditioned controls. Furthermore, Song *et al.*^33^ reviewed the differential expression of miRNAs in ischemic heart disease. To the best of our knowledge, there are no miRNA microarray analyses that have identified global changes in miRNA expression in the intestines of mice subjected to I/R injury that can be used to characterize the potential roles of miRNAs. In this study, we identified 19 miRNAs in the intestinal mucosa that changed by >1.5-fold relative to the sham group (Table 1). Similar to previous studies,^34^ we selected some miRNAs that were closely related to organs I/R injury. Among these differentially expressed miRNAs, let-7b, miR-26b, miR-182, miR-192, let-7d, miR-15b, miR-16, let-7a and miR-378, were confirmed by RT-qPCR to exhibit marked decreases in expression in the intestinal mucosa following intestinal I/R (Table 2). Previous studies have demonstrated the role of miR-378 in the regulation of cell apoptosis. For example, a recent study demonstrated that the downregulation of miR-378 supports cell survival by targeting the insulin-like growth factor receptor in cardiomyocytes and acts as a negative regulator.^35^ Paradoxically, some researchers have found that miR-378 may suppress luteal cell apoptosis by targeting the interferon gamma receptor 1 gene,^36^ and the overexpression of miR-378 attenuates I/R-induced cell apoptosis by inhibiting caspase-3 expression in cardiomyocytes.^37^ Therefore, the role of miR-378 in the regulation of intestinal epithelial cell death remains an enigma. Thus, we selected miR-378 for further functional studies.

Chemically modified agomir and antagomir have been used to increase or decrease miRNA expression *in vivo*.^38^ Therefore, we first used agomiR-378 and antagomiR-378 and found that they significantly increased and decreased miR-378 expression in the intestinal mucosa, respectively (Figure 2a). Furthermore, when pretreated with agomiR-378, intestinal I/R injury was attenuated, as evidenced by the significantly decreased Chiu’s scores, DAO activities and TUNEL-positive epithelial cells. By contrast, miR-378 antagomir pretreatment aggravated the intestinal tissue injury (Figures 2b and f). Obviously, the current results indicated that miR-378 may have a protective role in intestinal I/R-induced intestinal injury.

MiR-378 is predicted to have many potential targets.^39, 40^ More importantly, these assumed targets include both well-characterized pro-apoptotic and anti-apoptotic targets. Therefore, it is necessary to evaluate the long-term and global consequences of miR-378 overexpression in adult mice. To generate miRNA-378 overexpression TG mice, the specific promoter and pronuclear injection were used to drive the expression of miR-378 in mice. RT-qPCR analysis revealed that miR-378 was successfully overexpressed in TG mice to a level of 3.39-fold that observed in WT mice (Figure 3a). Thereafter, we performed further biochemical and physiological studies. As demonstrated, miR-378 overexpression in TG mice alleviated intestinal I/R injury (Figures 3b and f). OGD/R served to create an *in vitro* model of intestinal I/R injury that has previously been proven to be more amenable to the molecular dissection of cell death mechanisms.^41^ Consistent with evidence from the intestinal protection *in vivo*, our *in vitro* data revealed that IEC-6 cell viability was significantly preserved after transfection with the miR-378 mimic, whereas OGD/R challenge led to obvious cell death. By contrast, the IEC-6 cell viability was further decreased compared with the OGD/R group following pretreatment with the miR-378 inhibitor (Figure 4b). Taken together, our results highlight the crucial role of miR-378 in reducing the intestinal injury induced by I/R.

Previous studies have documented that apoptosis is the main mode of intestinal mucosal cell death after intestinal I/R,^8, 9^ and the exploration of the mechanisms of apoptosis might lead to effective therapy for organ I/R injury.^5, 42, 43^ Therefore, we investigated the role of miR-378 in the regulation of intestinal epithelial cell apoptosis. In this study, we found that the intestinal mucosal cell apoptotic index was reduced following pretreatment with miR-378 agomir, whereas miR-378 antagomir administration increased the apoptotic index. Consistent with the evidence of intestinal protection *in vivo*, our *in vitro* data demonstrated that the intestinal apoptotic/necrotic cells were also markedly reduced when pretransfected with miR-378 mimic in the OGD/R-challenged IEC-6 cells, whereas miR-378 inhibitor transfection aggravated the apoptotic and necrotic cells (Figure 4d). These data indicate that increased expression of miR-378 exerts anti-apoptotic properties. As intestinal epithelial cell apoptosis has been known to contribute to intestinal I/R injury, and a reduction in intestinal mucosa apoptosis could reduce the intestinal injury induced by I/R, it can be concluded that the overexpression of miR-378 attenuated intestinal I/R injury by inhibiting intestinal epithelial cell apoptosis.

The regulation of miRNAs and their interactions is complex. It is well accepted that an miRNA can bind to a large number of mRNAs in a manner dependent on a signature sequence in the 3’-UTR region of the target mRNAs. A specific mRNA can be controlled by many miRNAs if the miRNAs contain complementary binding sites. Structural studies have estimated that most miRNAs can inhibit approximately 200 mRNAs.^44^ Although our study has identified a relationship between miR-378 and its target caspase-3 in the ischemic intestine using prediction algorithms (i.e., TargetScan, miRanda, CLIP-Seq and miRDB) and the dual-luciferase reporter assay, the protection afforded by the miR-378 mimetic is only partial, and therefore, other miRNAs and mRNAs might also have a role in the damage-sparing mechanism.

Our observation is consistent with recent reports that have demonstrated that miR-378 targets cleaved caspase-3. Wang *et al.*^45^ reported that miR-378 inhibits cell growth and enhances L-OHP-induced apoptosis in human colorectal cancer. Lee *et al.*^46^ found that miR-378 transfection could enhance cell survival and could reduce caspase-3 activity by inhibiting the expression of suppressor of fused and Fus-1. Caspase-3 is a well-established executor of apoptosis that acts by cleaving various substrate proteins and also amplifies the death signal from the plasma membrane by activating additional caspases. Our previous studies demonstrated that inhibiting the activation of caspase-3 could reduce ischemic intestinal injury. In this study, we found that intestinal I/R caused severe intestinal injury accompanied by a significant magnitude of miRNA expression, and overexpression of miR-378 decreased caspase-3 activation and reduced intestinal mucosa cell apoptosis/necrosis both *in vivo* and *in vitro*.

There are issues that remain to be resolved in this study. First, apoptosis is an ATP-dependent form of cell death program that is related to mitochondrial dysfunction and caspase activation. Our results showed that, in miRNA-378 overexpressing TG mice, the addition of agomiR-378 and miR-378 mimic decreased caspase-3 cleavage. However, whether miR-378 changes the mitochondrial function or ATP biogenesis remains to be determined in the future. Second, because several miRNAs responded rapidly to intestinal I/R, this study only investigated the function of miR-378 in ischemic intestine; whether other differentially expressed miRNAs (miR-182, miR-192, let-7a, etc.) have roles during the process was not explored. In addition, the parallel existence of necroptosis,^41^ apoptosis and autophagy of epithelial cells following ischemic stimulus demonstrates the complexity of the pathophysiology of intestinal I/R, and the regulatory process by which miRNAs are differentially expressed requires further exploration.

In summary, the modulation of miRNA expression offers a potential therapeutic option for intestinal I/R injury. This study was the first to analyze miRNA expression profiles in the intestinal mucosa after intestinal I/R and to find that miR-378 is significantly downregulated during the process. Further studies revealed that increased expression of miR-378 attenuated intestinal I/R injury by inhibiting intestinal mucosal cell apoptosis, which is associated with the regulatory effects of miR-378 on caspase-3 signaling. The protection rendered by miR-378 may represent a potential novel therapeutic target for the treatment of diseases related to intestinal I/R injury.

## Materials and methods

### *In vivo* experiments

#### Animal model and preparation of specimens

All studies were approved by the Animal Care Committee of Sun Yat-sen University (China) and were performed in accordance with National Institutes of Health guidelines for the use of experimental animals. Ninety-six adult pathogen-free male mice (weighing 25–30 g) were housed in individual cages in a temperature-controlled room under a fixed circadian rhythm with free access to food and water.

Male FVB/N mice (8–10 weeks old) were anesthetized with pentobarbital (30 mg/kg, intraperitoneal injection). The small intestine was exteriorized by midline laparotomy. The intestinal I/R injury was established by occluding the superior mesenteric artery (SMA) with a microvessel clip for 60 min followed by 120 min of reperfusion according to our previous study.^9^ Ischemia was recognized by the existence of pulselessness or a pale color of the small intestine. The return of pulses and the re-establishment of the pink color were assumed to indicate valid reperfusion of the intestine. At the end of the reperfusion, a segment of 10 cm of the intestine was cut 5 cm away from the ileocecal valve and was divided into two segments. The segments were fixed in 10% neutral formaldehyde, paraffin embedded for morphological analysis and washed with cold saline after being scraped off. The intestinal mucosa was dried with filter paper and preserved at –80 °C for detection.

#### Histological assessments of intestinal injury

The segment of small intestine was stained with hematoxylin and eosin. Damage of intestinal mucosa was evaluated independently by two pathologists who were blinded to the study groups. The degree of injury was evaluated using the criteria of Chiu's score as previously described.^47^ A minimum of five randomly chosen fields from each mouse were evaluated and averaged to determine mucosal damage.

#### Detection of DAO activity in the plasma

To further confirm intestinal injury, serum DAO, a sensitive marker that reflects small intestinal mucosal injuries, was detected using a chemical assay kit (Nanjing Jiancheng Biochemicals Ltd, Nanjing, China) with an ultraviolet spectrophotometer at the wavelength of 436 nm according to the manufacturer’s protocol.

#### MiRNA microarray analysis

Mice were randomly assigned to the sham or the injury group (*n*=5 per group). Total miRNAs were isolated from the intestinal mucosa and processed for miRNA microarray analysis using the miRCURY LNA Array (version 11.0 Exiqon A/S, Vedbaek, Denmark) system. RNA samples were labeled with an ExiqonmiRCURY Hy3/Hy5 power labeling kit and hybridized on a miRCURY LNA Array station. Scanning was performed with an Axon GenePix 4000B microarray scanner. GenePix pro version 6.0 was used to read the raw image intensities. The intensity of the green signal was calculated after background subtraction, and replicated spots on the same slide were averaged to obtain median intensity. The median normalization method was used to acquire normalized data (foreground minus background divided by median). The median was the 50th percentile of miRNA intensity and was >50 in all samples after background correction. The threshold value for significance used to define the upregulation or downregulation of miRNAs was a *P*-value <0.05 calculated by *t*-test. The miRNAs that were selected for investigation in our study were further filtered on the basis of expression levels and previously published data.

#### Prediction of miRNA-mRNA targets

MiRNA targets are difficult to identify because of the lack of strict base pairing between miRNA and mRNA target sequences. Several computation algorithms, including TargetScan,^48^ miRanda,^49^ CLIP-Seq^50^ and miRDB,^51^ aid this task by examining base-pairing rules between miRNAs and the locations of mRNA target sites with binding sequences within the target’s 3’-UTR and the conservation of target binding sequences within related genomes. After all the predicted targets were identified, we further calculated the intersection of the above four algorithms and drew a Venn diagram to analyze the specific novel candidate predicted targets (Supplementary Figure 1). Genes that were predicted by three of the TargetScan, miRanda, CLIP-Seq and miRDB algorithms were regarded as potential targets of a certain miRNA.^52^

#### *In vivo* administration of agomiR-378 and antagomiR-378

To investigate the effects of miRNA-378 following intestinal I/R injury, the chemically modified agomir and antagomir were used to increase or decrease miRNA expression *in vivo*.^38^ The miRNA agomir is a chemically modified, cholesterylated, stable miRNA mimic, and its *in vivo* delivery resulted in target silencing similar to the effects induced by the overexpression of endogenous miRNA. The miRNA antagomir is a chemically modified, cholesterol-conjugated, single-stranded RNA analog that complements the miRNAs and could efficiently and specifically silence the endogenous miRNA. AgomiR-378 and antagomiR-378 were synthesized by RiboBio (Guangzhou, China). Scrambled mimics that did not target any miRNA were injected as a NC. Their sequences are listed as follows: agomir-378: 5′-ACUGGACUUGGAGUCAGAAGG-3′, 3′-UGACCUGAACCUCAGUCUUC-5′ agomir-378 NC: 5′-UCACAACCUCCUAGAAAGAGUAGA-3′, 3′-AGUGUUGGAGGAUCUUUCUCAUCU-5′, antagomir-378: 5′-CCUUCUGACUCCAAGUCCAGU-3′ and antagomir-378 NC: 5′-CAGUACUUUUGUGUAGUACAAA-3′.

The mice received either agomir, antagomir or their NCs (100 *μ*l) via tail vein injection (40 mg/kg body weight, *n*=8 per group) for three consecutive days. Expression of miR-378 was detected on the fourth day by RT-qPCR.

#### Generation of a miR-378 TG mouse model

MiRNA-378-overexpressing TG (Cyagen Biosciences, Guangzhou, China) mice were produced by pronuclear injection. Briefly, we first acquired the genomic sequence of miR-378 from the Origene company (OriGene Technologies, Rockville, MD, USA) website, then intercepted Villin genes upstream of the transcription start 7-kb site and the first intron 5.5 kb of sequence based on previous studies. We next added the linearization restriction site *Mlu*I between the two homology arms of the inserted vector pStar-K using Gap-repair ways to obtain a final vector. To generate miRNA-378-overexpressing TG mice, the specific promoter and pronuclear injection were used to drive the expression of miR-378 in founder mice. The offspring of FVB/N mice propagation was based on founder FVB/N mice using full-sib mating. The level of miR-378 was detected in the third-generation mice by RT-qPCR, normalized to U6, and expressed as the fold change relative to the WT control. The primer designs and sequential methods are listed as follows: Primer design: homologous arm before PCR amplification: *Bam*HI-upstream-F: 5′-ACTCGCGGATCCTTTAATCCCATCACTTGGGAGG-3′, *Mlu*I-upstream-R: 5′-GCGTAGTCCCATCTGGGAAATACGCGTGGCAATGGCAGAGTGAAGAG-3′. Homologous arm after PCR amplification: *Mlu*I-upstream-F: 5′-ACGCGTATTTCCCAGATGGGACTACGC-3′, downstream-DsRed2-R: 5′-GACGTTCTCAGTGCTATCCATGGTGGCTGGGGGTCTTGACCACTGTAG-3′. DsRed2 amplification: Downstream-DsRed2-F: 5′-GCCACCATGGATAGCACTGAGAACGTC-3′, DsRed2-mir-378-R: 5′-CACTGCTTCTGCTGACAACTGCTACTGGAACAGGTGGTGG-3′. Mir-378 amplification: DsRed2-mir-378-F: 5′-GTTCCAGTAGCAGTTGTCAGCAGAAGCAGTG-3′, NotI-mir-378-R: 5′-AAGGAAAAAAGCGGCCGCCTGGGTTAGCCACCAAAGAC-3′, Vector of pStar-K for subsequent restructuring: pStar-K-F: 5′-ATTTCCCAGATGGGACTACGC-3′, pStar-K-R: 5′-GGCAATGGCAGAGTGAAGAG-3′.

#### Real-time quantitative polymerase chain reaction (RT-qPCR)

Total RNA from fresh tissues and cell lines was extracted using Trizol Reagent (Invitrogen, Grand Island, NY, USA) following the manufacturer's instructions. The quality of RNA was examined using a UV-Vis spectrophotometer UV-1800 (Shimadzu, Japan). RNA integrity was verified using 1.5% agarose gel electrophoresis with OD260/280 between 1.8 and 2.0, and RNA 28 s/18 s>1. RT-qPCR analysis of the miRNA was performed using RT primer and TaqMan probe for the miRNAs (Ribobio) on an ABI 7500 (Applied Biosystems, Foster City, CA, USA) according to the manufacturer's protocol. Their primers are listed in Supplementary Table 1. The expression of RNA U6 small nuclear 2 (RNU6B) was used as an endogenous reference control. Each RT-qPCR analysis was repeated twice using three independent specimens. The relative abundance of miRNA in the tissues and cell lines was calculated using the equation RQ=2^− ΔΔCT^.

#### *In situ* TUNEL for intestinal mucosa apoptosis assay

The ileal fragments were fixed in 10% neutral formaldehyde and embedded in paraffin. The apoptosis of the intestinal mucosal epithelial cells was performed with the terminal deoxynucleotidyltransferase (TdT)-mediated dUDP-biotin nick end labeling (TUNEL) method as previously described. Cell death was assessed using an *In Situ* Detection assay kit (Roche, Indianapolis, IN, USA). TUNEL-positive cells were characterized by dark brown staining of the nucleus and nuclear membrane. Quantitation was performed by counting the numbers of positive cells in five randomly chosen fields within each slide at 400 × independently by two pathologists who were blinded to the study groups. The rate of cell apoptosis (apoptotic index) is expressed as a percentage of the TUNEL-positive cells using the following formula: the number of TUNEL-positive cell nuclei/the number of total cell nuclei × 100.

### *In vitro* experiments

#### IEC-6 cell culture and oxygen and glucose deprivation/reperfusion (OGD/R) model

Intestinal epithelial cells (IEC-6, catalog no. RL-1592) were obtained from the American Type Culture Collection (ATCC, Manassas, VA, USA) and were cultured in Dulbecco’s modified Eagle’s medium containing 4.5 g/l d-glucose, 10% v/v fetal bovine serum (FBS) and 1% penicillin/streptomycin antibiotics (Gibco, Invitrogen Ltd, Shanghai, China). The cells were maintained under standard cell culture conditions at 37 °C under 5% CO_2_ and 21% O_2_. To simulate the situation of intestinal ischemia *in vivo*, oxygen and glucose deprivation (OGD) was used *in vitro*. Briefly, after the IEC-6 cells were grown under normoxic conditions up to a confluence of 80%, OGD was induced by exchanging the normoxic medium with D-Hanks buffer (OGD medium), and the cells were then switched to a modular incubator chamber filled with a 95% N_2_ and 5% CO_2_ gas mixture for 4 h at 37 °C. Following the OGD, the medium was changed back to the normoxic medium, and the cells were incubated under the normal conditions for an additional 4 h of reoxygenation.^53, 54^ The cells that were not subjected to OGD/R were incubated at 37 °C with 5% CO_2_ for the same time periods and served as control (sham). Six independent *in vitro* experiments were performed in the present work.

#### miRNA transfection

To assess the function of elevated levels of miR-378 in IEC-6 cells, pre-miR-378 (mimic) and miR-378 inhibitor were transfected for miR-378 overexpression and inhibition, respectively. Before large-scale transfection, the conditions were optimized with cells cultured in 96-well plates in Opti-MEM-I serum-reduced medium, NC oligonucleotide with FAM moiety at the 5’ end, and Lipofectamine 2000 Reagent (Invitrogen, Carlsbad, CA, USA) following the manufacturer’s procedure. The transfection efficiency was determined by the use of the NC oligonucleotide with 6-FAM at the 5’ end. After optimization, transfection complexes were added to the cells at a final oligonucleotide concentration of 50 nM.^55^ MiR-378 mimic and inhibitor NCs were also involved in this study. All oligonucleotides were purchased synthesized (RiboBio). All experiments were performed in triplicate. After 48 h transfection, the cells were harvested and used for molecular and cellular studies.

#### Dual-luciferase reporter analysis

293 T cells (5 × 10^4^cells/well) were cultured in 48-well plates for 24 h and then transfected with PMIR-RB-REPORT-caspase-3-3’-UTR (1 *μ*g/well) (RiboBio) and miR-378 mimic (100 nm per well) or a NC, separately. The plasmid (PMIR-RB-REPORT-caspase-3-3’-UTR) contained a synthetic Firefly luciferase gene that served as the reference gene and caspase-3 3’-UTR downstream from Renilla luciferase served as a reporter gene. The cells were harvested 24 h after transfection, and the ratio of Renilla and Firefly luciferase activities was measured with a dual-luciferase reporter assay kit (Promega, Madison, WI, USA) according to the manufacturer’s instructions.

#### Cell viability assessment

The cells (1 × 10^5^) were plated into each of the wells of the 96-well plates. After OGD/R, the MTT assay was used to detect the IEC-6 cells viabilities. Briefly, MTT (3-(4,5-dimethylthiazol-2-yl)-2,5-diphenyl-2-H-tetrazolium bromide, 5 mg/ml in phosphate-buffered saline, Sigma, Shanghai, China) was added to each well and incubated for 4 h at 37 °C. Then, the medium was replaced with 150 *μ*l buffered DMSO. The optical density (OD) was recorded using a microplate reader at the wavelength of 490 nm. Cell viability is expressed as a percentage of the sham value.

#### Flow cytometric analysis

IEC-6 cell apoptosis was assayed by flow cytometry according to the protocol provided by the manufacturer after OGD/R. Briefly, the cells were washed twice with cold PBS before staining with FITC Annexin V and propidium iodide (PI) using the Annexin V-FITC Apoptosis Detection Kit I (BD Biosciences, Franklin Lakes, NJ, USA) for 15 min at room temperature in the dark. The stained cells were analyzed using flow cytometry within 1 h. The FITC Annexin V^+^/PI^-^and FITC Annexin V^+^/PI^+^ cell populations were considered to represent necrotic and apoptotic cells.

#### Western blot analysis

Protein was extracted from intestinal mucosal samples and IEC-6 cells. The levels of cleaved caspase-3 expression were determined using a specific antibody against the large fragment (17 kDa) of activated caspase-3 that resulted from cleavage. Twenty micrograms of total protein was electrophoresed on a 12% (w/v) SDS-PAGE gel, transferred onto a nitrocellulose membrane and blocked with 5% (w/v) nonfat dried milk. The membranes were probed with the caspase-3 primary antibody (Cell Signaling Technology, Danvers, MA, USA, 9665, 1 : 200) followed by the peroxidase-conjugated secondary antibody. The protein signal was visualized with chemiluminescence reagents under a GeneGnome Bio Imaging System (Syngene, MD, USA). The amount of cleaved caspase-3 was quantified by densitometry and normalized to the internal control (GAPDH).

### Statistics analysis

All data are expressed as the mean±S.D. Differences between two groups were analyzed by Student’s *t*-test and between multiple groups by a one-way ANOVA *post hoc* procedure. *P*<0.05 (two-sided tests) was considered statistically significant.

## Figures and Tables

**Table 1:** miRNAs analyzed by microarray

**Name**	**Fold change injury *versus* sham**	***P*****-value**	**Genomic coordinates**
mmu-miR-292-3p	2.079233	0.049315	chr7: 3 219 189–3 219 270
mmu- miR-103	0.419844	0.037357	chr11: 35 782 396–35 782 481
mmu-let-7b	0.379569	0.040591	chr15: 85 707 319–85 707 403
mmu-miR-151	0.298605	0.048223	chr15: 73 254 815–73 254 882
mmu-miR-352	0.370564	0.046623	&
mmu-miR-26b	0.317149	0.02567	chr1: 74 394 310–74 394 394
mmu-miR-182	0.417398	0.007855	chr6: 30 165 918–30 165 992
mmu-miR-31*	0.248849	0.005678	chr4: 88 910 557–88 910 662
mmu-miR-192	0.317994	0.005864	chr19: 6 264 844–6 264 932
mmu-let-7d	0.474344	0.026367	chr13: 48 536 012–48 536 114
mmu-miR-429	0.251106	0.013855	chr4: 156 053 905–15 6053 987
mmu-miR-32	0.317152	0.036267	chr4: 56 895 229–56 895 298
mmu-miR-192*	0.267506	0.022734	chr19: 6 264 844–6 264 932
mmu-miR-96	0.25604	0.023989	chr6: 30 169 446–30 169 551
mmu-miR-15b	0.307694	0.021726	chr3: 69 009 772-–69 009 835
mmu-miR-16	0.349734	0.041569	chr14: 61 631 880–61 631 972
mmu-let-7a	0.434087	0.047695	chr13: 48 538 179–48 538 272
mmu-miR-30b-5p	0.425581	0.035156	chr15: 68 337 415–68 337 510
mmu-miR-378	0.40383	0.003153	chr18: 61 397 835–61 397 900

&: only experimental evidence. Not found in mouse genome. Confidence:undefined.

Microarray analysis identified 19 differentially expressed miRNAs in response to intestinal I/R, all of which exhibited a *P*-value <0.05 calculated by *t*-test when compared with the sham group (*n*=5 per group)

**Table 2:** Intestinal I/R-responsive miRNA candidates were confirmed by RT-qPCR

**miRNA**	**Relative expression** ( 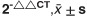 )		***P*****-value**	**Fold change**
	**Sham group**	**Injury group**		
mmu-miR-26b	0.98±0.02	0.637±0.06	0.001	1.54
mmu-miR-16	1.48±0.42	0.38±0.06	0.011	3.90
mmu-let-7b	1.02±0.06	0.30±0.05	0.001	3.40
mmu-miR-103	1.00±0.2	0.35±0.05	0.06	2.86
mmu-miR-15b	0.85±0.19	0.35±0.09	0.015	2.43
mmu-miR-192	0.96±0.04	0.22±0.06	0.001	4.36
mmu-miR-182	0.87±0.14	0.13±0.02	0.001	6.69
mmu-let-7a	1.08±0.08	0.21±0.04	0.001	5.14
mmu-miR-378	0.88±0.17	0.26±0.06	0.04	3.38

A fold change of at least 1.5 and putative target mRNAs that were linked to cell death were identified (*n*=5 per group)

**Table 3:** Predicted targets of miR-378

**GeneSymbol**	**TargetScan**	**miRanda**	**CLIP-Seq**	**miRDB**
ANKRD52	1	1	1	0
ARF2	0	1	1	1
ARRDC2	1	1	0	1
BCL2L2	1	1	1	0
CAMK2N1	0	1	1	1
CCDC142	0	1	1	1
CLCN4-2	0	1	1	1
CLOCK	0	1	1	1
CSNK1G1	1	1	0	1
DNAJA2	0	1	1	1
DYRK1A	1	1	1	0
EFNA5	1	1	0	1
FOXG1	1	1	0	1
FRMPD4	1	1	1	0
GPM6B	1	1	1	0
GRB2	1	1	0	1
GRSF1	1	1	0	1
H3F3B	1	1	1	0
KPNA6	1	1	1	0
LASS6	0	1	1	1
MAPK1	1	1	1	0
MPP3	0	1	1	1
MTSS1L	1	1	1	1
NEUROD1	1	1	1	1
NMNAT2	0	1	1	1
NPAS4	1	1	0	1
PAPOLA	1	1	0	1
PDE7B	1	1	0	1
PGRMC1	0	1	1	1
CASPASE-3	1	1	1	1
PSD3	1	1	0	1
SLC2A3	1	1	1	0
SLC9A5	1	1	0	1
SPEG	1	1	0	1
SULF1	1	1	0	1
SYN2	0	1	1	1
TBC1D22A	0	1	1	1
TLK2	1	1	0	1
UGCG	0	1	1	1
VAT1L	1	1	1	0

1: existent, 0: non-existent
