# Supplementary material for: MicroRNA-378 protects against intestinal ischemia/reperfusion injury via a mechanism involving the inhibition of intestinal mucosal cell apoptosis
Source: Cell Death Dis. 2017 Oct 12;8(10):e3127–. doi: 10.1038/cddis.2017.508 (PMC5682673; doi:10.1038/cddis.2017.508)

Supplementary Materials

Supplementary Table 1:

Primers for real-time quantitative polymerase chain reaction.

Gene	Bidirectional primer sequence
U6	F:5’GCTTCGGCAGCACATATACTAAAAT3’
	R:5’CGCTTCACGAATTTGCGTGTCAT3’
miR-16	F:5'GGGTAGCAGCACGTAAATA3'
	R:5'CAGTGCGTGTCGTGGAGT3'
mir-15b	F: 5'GGGGTAGCAGCACATCATG3'
	R:5’GTGCGTGTCGTGGAGTCG 3’
miR-378	GSP:5’GGACACTGGACTTGGAG3’
	R:5’TGCGTGTCGTGGAGTC3’
miR-32	F:5’GGGGCTATTGCACATTACTA3’
	R:5’CAGTGCGTGTCGTGGAG3’
miR-103	F:5’GAGAGAGCAGCATTGTACAG3’
	R:5’CAGTGCGTGTCGTGGA3’
miR-182	F:5'GCTTTGGCAATGGTAGAAC3’
	R:5’CAGTGCGTGTCGTGGAG3’
miR-192	F:5'GGGCTGACCTATGAATTG3’
	R:5’TGCGTGTCGTGGAGTC3’
miR-26b	F:5'GGGGTTCAAGTAATTCAGG3’
	R:5’TGCGTGTCGTGGAGTC3’
let-7b	F:5'GGGGTGAGGTAGTAGGTTG3’
	R:5’TGCGTGTCGTGGAGTC3’
7a	F:5'GGGGTGAGGTAGTAGGTTGT3'
	R:5'CAGTGCGTGTCGTGGAGT3'

Supplementary Figure 1: Venn diagram analysis of MiRNA-378 predicted putative targets.

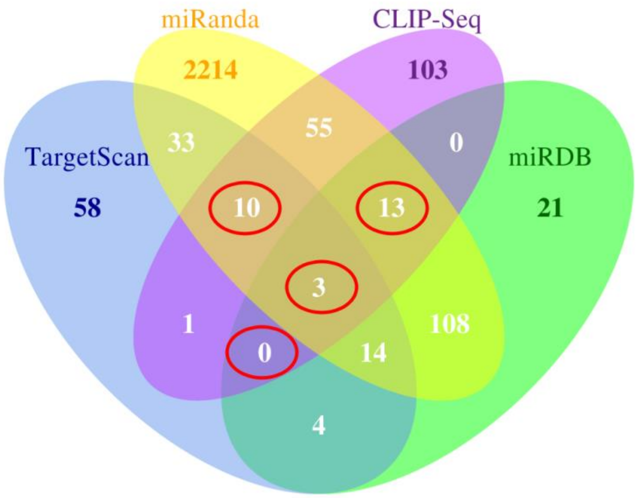

Supplement: Supplementary Materials [file cddis2017508x1.pdf]
